# Ibrutinib ameliorates cerebral ischemia/reperfusion injury through autophagy activation and PI3K/Akt/mTOR signaling pathway in diabetic mice

**DOI:** 10.1080/21655979.2021.1974810

**Published:** 2021-10-04

**Authors:** Lei Jin, Yun Mo, Er-Li Yue, Yuan Liu, Kang-Yong Liu

**Affiliations:** a Department of Neurology, Shanghai University of Medicine & Health Sciences Affiliated Zhoupu Hospital, Shanghai, 201318, PR China; b Department of Neurology, Guizhou Medical University, Guiyang, Guizhou, 550025, PR China

**Keywords:** Ibrutinib, cerebral ischemia/reperfusion injury, diabetes, autophagy, pi3k/akt/mTOR pathway

## Abstract

Bruton’s tyrosine kinase (BTK) is involved in the diabetogenic process and cerebral ischemic injury. However, it remained unclear whether BTK inhibition has remedial effects on ischemia/reperfusion (I/R) injury complicated with diabetes. We aim to investigate the regulatory role and potential mechanism of ibrutinib, a selective inhibitor of BTK, in cerebral I/R injured diabetic mice. The cytotoxicity and cell vitality tests were performed to evaluate the toxic and protective effects of ibrutinib at different incubating concentrations on normal PC12 cells or which were exposed to high glucose for 24 h, followed by hypoxia and reoxygenation (H/R), respectively. Streptozotocin (STZ) stimulation-induced diabetic mice were subjected to 1 h ischemia and then reperfusion. Then the diabetic mice received different dosages of ibrutinib or vehicle immediately and 24 h after the middle cerebral artery occlusion (MCAO). The behavioral, histopathological, and molecular biological tests were then performed to demonstrate the neuroprotective effects and mechanism in I/R injured diabetic mice. Consequently, Ibrutinib improved the decreased cell viability and attenuated oxidative stress in the high glucose incubated PC12 cells which subjected to H/R injury. In the I/R injured diabetic mice, ibrutinib reduced the cerebral infarct volume, improved neurological deficits, ameliorated pathological changes, and improved autophagy in a slightly dose-dependent manner. Furthermore, the expression of PI3K/AKT/mTOR pathway-related proteins were significantly upregulated by ibrutinib treatment. In summary, our finding collectively demonstrated that Ibrutinib could effectively ameliorate cerebral ischemia/reperfusion injury via ameliorating inflammatory response, oxidative stress, and improving autophagy through PI3K/Akt/mTOR signaling pathway in diabetic mice.

## 1. Introduction

Stroke is a major cause of disability, and has been one of the most referenced causes of mortality worldwide. Ischemic stroke and hemorrhagic stroke accounted for approximately 80% and 20%, respectively, of stroke cases [1]. Thus, reducing brain injury and improving the prognosis of ischemic stroke is becoming an urgent neurology problem. Current therapeutic approaches for ischemic stroke are limited to thrombolysis, represented in tissue plasminogen activator (tPA). Lamentedly, the limited window for tPA could only benefit a small part (3–5%) of patients [2]. Diabetes aggravates cerebral vessels’ susceptibility and vulnerability, which increases an immense risk for ischemic stroke. Stroke has become a serious death threat for diabetic patients. The proportion of stroke in diabetic patients is higher than that in healthy people, and the prognosis and mortality after stroke are not optimistic [3].

Diabetes increases stroke risk of 2 – to 5-fold, especially ischemic stroke [4]. Unfortunately, the most effective treatment of stroke by tPA in patients with diabetes prompts increased morbidity of intracerebral hemorrhage and the following neurological deficits than regular patients [5]. Newly developed treatments for regular stroke patients did not achieve their desired effect in diabetic stroke patients [6]. In addition, the study by Liu et al. revealed the molecular regulatory mechanism of neuronal apoptosis under the cerebral ischemia/reperfusion injury and demonstrated that miR-484 alleviates cerebral ischemia/reperfusion injury-induced neuronal apoptosis in mice by targeting apoptosis facilitator BCL2L13 [7]. However, the therapeutic modalities for specific and efficient treatment against diabetic stroke did not involve. Therefore, exploring the vital pathological process in diabetes complicated with stroke carries significant implications for developing new therapeutic strategies.

Autophagy is a self-eating process. At this state, a phagophore can elongate and engulf to enclose cytoplasmic contents into an autophagosome, which eventually targets the lysosomes. Autophagy is momentous for maintaining cell homeostasis, promoting cell development, and accelerating nutrient recycling [8]. Under a condition of stress stimuli, such as hypoxia, nutrient deprivation, DNA damage, oxidative stress, autophagy supplies energy for vital cellular metabolic activity and cellular survival [9]. Diabetes increases oxidative stress, diminishes autophagy, and aggravates cerebral ischemic injury in animal models [10]. Autophagic dysfunction is a pathological feature of ischemia/reperfusion (I/R) injury [11]. Researches showed that boosting autophagy could modify ischemic brain injury in diabetic rats [12].

Ibrutinib is a selective covalent Bruton’s tyrosine kinase (BTK) inhibitor, which could penetrate blood-brain barrier [13] and approved by the Food and Drug Administration (FDA) in 2013 as a treatment of several hematological malignancies. BTK is a critical component of B-cell receptor signaling and showed a more general role in immune regulation and function [14]. Ibrutinib can act through various aspects and has been proven to be a promising new drug. Autophagy is negatively regulated by the mammalian target of rapamycin (mTOR) complex 1 [15]. Downstream of the BTK signaling pathway, for example, the PI3K/Akt pathway, regulates autophagy, showing a possible link between ibrutinib and autophagy. Besides, studies indicated that inhibiting BTK could be a potent therapeutic target in ischemic stroke [16], and modulation of signaling via BTK prevented T cell-mediated autoimmune diabetes [17]. Even so, there is no relevant research on whether ibrutinib regulates autophagy and acts as therapy for cerebral I/R injury in the diabetic state.

In this study, we hypothesized that ibrutinib treatment may trigger beneficial effects in cerebral I/R injury in diabetic mice. To prove such a hypothesis, we evaluated the effects of ibrutinib on ischemic brain injury, neurological function, inflammatory response, oxidative stress and autophagy in cerebral I/R injury in diabetic mice. In addition, the functional relevance of PI3K/Akt/mTOR signaling pathway in activated autophagy was also investigated. Our study demonstrated that in diabetes combined with ischemic stroke, ibrutinib administration strongly protected the cell and brain tissue injury, inhibited oxidative stress, and improved autophagy through signaling PI3K/Akt/mTOR pathway. Therefore, our research pointed out that ibrutinib can treat cerebral I/R injury complicated with diabetes. More importantly, we further probed ibrutinib’s potential mechanism as a therapeutic strategy in diabetes complicated with cerebral I/R injury, aiming to find a novel treatment for this complex disorder at the clinical stage.

## 2. Materials and methods

### 2.1 Animals

Eight-week male C57BL/6 mice weighed 22–24 g were obtained from Vitalriver (Beijing, China). The mice were grouped housed 5 per cage under standard conditions and maintained on a regular 12-hour light/dark cycle. All animal procedures were reviewed and approved by the ethics committee with the approval number of ATX2850201.

### 2.2 Cell culture and MTT assays

PC12 cells cultures were performed as previously described [18]. The cells were maintained in RPMI 1640 medium, including 10% fetal bovine serum and 100 μg/ml penicillin-streptomycin. PC12 cells were divided into five groups: 25 mmol/L high glucose-stimulated control (the HG group), high glucose-stimulated and H/R treated cells (the HH/R group), and high glucose-stimulated and 1 μmol/L, 3 μmol/L, and 5 μmol/L ibrutinib incubated cells subjected to H/R (the HH/R-Ib 1 μmol/L, HH/R-Ib 3 μmol/L, and HH/R-Ib 5 μmol/L group). H/R was induced after 3 h and 6 h hypoxia (95% N_2_ and 5% CO_2_) and reoxygenation (21% O_2_, 5% CO_2_ and 74% N_2_). Ibrutinib was administrated 6 h before H/R.

Cytotoxicity of ibrutinib was measured by MTT assays (MTT kit, Sigma-Aldrich, Oakville, ON, Canada). After the addition of 20 mL of MTT (5 mg/ml), PC12 cells were incubated for 4 h, then centrifuged at 1800 g for 5 min at 4°C, and cell viability was terminated by adding 150 μL buffered DMEM and shaking. The results were detected by a microplate reader at 490 nm.

### 2.3 Establishment of STZ induced diabetic mice model with cerebral I/R injury

Mice were allowed to acclimate to the environment of a well-ventilated room for a week before the experiment. Mice were intraperitoneally injected with 40 mg/kg STZ (Sigma-Aldrich, Oakville, ON, Canada) in 100 mol/L citrate buffer for a consecutive five-day schedule to induce type 1 diabetes. Blood glucose levels after STZ treatment were measured by FreeStyle Optium Neo meter. After 72 hours of STZ injection, those mice with fasting blood glucose value higher than 250 mg/dL were defined as diabetic state and used for the experiment. The health status of all the mice was observed persistently and no mice died during the modeling. The body weight and food and water intake amount of each mouse were also recorded. All animals were divided into five group: (1) Diabetic sham-operated group (DS group); (2) Diabetic I/R injured group (DIR group); (3) Diabetic I/R injured group with 1 mg/kg ibrutinib treatment (DIR-Ib 1 mg/kg group); (4) Diabetic I/R injured group with 3 mg/kg ibrutinib treatment (DIR-Ib 3 mg/kg group); (5) Diabetic I/R injured group with 5 mg/kg ibrutinib treatment (DIR-Ib 5 mg/kg group).

The cerebral I/R injury was induced by inserting a thread embolism in the right-sided middle cerebral artery (MCA) of diabetic mice, as described previously with minor modifications [19]. Mice were anesthetized using an isoflurane gas system with 5% isoflurane and maintained at 1.5% isoflurane during surgery. The right MCA was exposed after ventral neck incision, and the arteries were isolated. A coated 6–0 filament (Covidien, Mansfield, MA, USA) was inserted into the internal carotid artery through the external carotid artery stump and gently advanced 11 mm past the carotid bifurcation to occlude the middle cerebral artery. Cerebral blood flow (CBF) was monitored by Doppler flowmetry (Moor Instruments, Devon, UK), and animals with less than 80% reduction in CBF were excluded. The filament was gently withdrawn for reperfusion after 60 min of blocking. Animals were maintained at a body temperature of 36.5–37.5°C during the operation using a heat lamp and a rectal thermometer. No mice died during or after MCA surgery. In addition, mice in diabetic sham-operated group (DS group) underwent the same procedure apart from filament insertion.

### 2.4 Behavioral tests

The neurological score was measured 3 days after reperfusion based on the neurological grading scale previously described [20]. The neurological score was calculated from 0 to 4, and higher scores indicate more severe neurological impairment. The scores were defined as the following rule: 0, normal; 1, a slight decrease in mobility and passivity; 2, medium neurological deficits; 3, handicapped with basic walking ability; 4, incapacity to move; 5, death. The scores were performed and analyzed by two independent investigators.

The adhesive removal test represented the sensorimoter function of each mouse. A small tape was stuck on the uninjured forepaw, and the tape removal time of each mouse was recorded in every trial for 120 s maximum. The final score was expressed as the mean time of each mouse over three repeated trials.

The beam walk test represented balance. Each mouse was trained for 3 trials before the formal experiment. Mice were placed on a high and narrow beam (5×1000 mm), and the time each mouse spent crossing the beam was recorded. The final score was expressed as the mean time of each mouse over three repeated trials.

The rotarod test represented motor coordination. Mice were placed on an accelerating rod (UGO Basile, Comerio, VA, Italy) that speed accelerated 4–40 rpm in 300 s. The final score was expressed as the mean time of the latency to fall of each mouse over three repeated trials.

### 2.5 TTC and H&E Staining

Triphenyltetrazolium chloride (TTC) staining was applied to measure the cerebral infarction [21]. After mice were sacrificed, brain tissues (coronal plane) were removed immediately and continuously cut into 2 mm intervals with a mold. Brain sections were stained in 1% TTC solution for 5 min and washed in 1 x PBS. The sections were photographed by a camera and analyzed by ImageJ software. The infarct size was calculated as the percentage of infarct area/total area. Hematoxylin-eosin (H&E) staining was performed on 5 μm slides of brain tissue using previously described procedure [22]. The H&E stain was examined using Slide-digital microscopy (Olympus, Tokyo, Japan).

### 2.6 TUNEL Assay

TUNEL staining was performed following the manufacturers’ instructions using a commercial kit (Promega Corp., WI, USA). The brain tissue sections permeabilized in Triton 100 for 15 min and then washed 3 times in 1 x PBS. After one-hour incubation at 37°C in the TUNEL reaction mixture and another 3 times wash, nuclei were counterstained with DAPI stain. The stained slides were observed through a slide scanner (APERIO CS2, Leica, Germany) and brown color indicates apoptotic cells.

### 2.7 ROS, MDA, and SOD Quantification

The reactive oxygen species (ROS) content in cell supernatant was determined by the DCFDA-Cellular ROS Detection Assay Kit (Abcam) according to the manufacturer’s instructions. Malondialdehyde (MDA) level and superoxide dismutase (SOD) activity in cell lysates were detected by ELISA kits (Krishgen Biosystems, USA). The amount of interleukin-1α (IL-1α), interleukin-6 (IL-6) and tumor necrosis factor α (TNF-α) in tissue homogenates was detected by ELISA kits (Krishgen Biosystems, USA). Mitochondria-generated ROS, MDA, and SOD of individual brain tissue samples were measured using the respective kits (Krishgen Biosystems, USA) according to the manufacturers’ instructions.

### 2.8 Western Blot

Western blot was performed according to a previously described protocol [14]. The ischemic brain tissues were homogenized and centrifuged then the supernatants were collected. Total protein content was determined by BCA assay (Beyotime, China) according to the manufacturers’ instructions. 20 µg protein of supernatant aliquots were run on SDS-PAGE (10%). Bovine serum albumin (3%) was blocked in 0.2% to 0.4% TBST for 60 min and incubated in the primary antibodies in 0.5% BSA in 0.1% TBST O/N at 4 °C. Primary antibodies used in this experiment were rabbit monoclonal antibodies following: LC3 (Abcam, USA), p62 (Cell Signaling Technology, USA), Atg7 (Beyotime, China), PI3K (1:1,000, Cell Signaling Technology, USA), mTOR (Abcam, USA), Phospho-mTOR (Abcam, USA), Akt (Cell Signaling Technology, USA), Phospho-Akt (Cell Signaling Technology, USA), and GAPDH (Abcam, USA). Next, a goat anti-mouse IgM (1:5,000) or antirabbit IgG (1:5,000) were incubated with the membranes for 1 h. The bands were semi-quantified by densitometry using ImageJ software.

### 2.9 Statistical Analysis

SPSS 17.0 software (Chicago, USA) was used for statistical analyzing. The quantification data were present as mean ± SD. Statistical data were analyzed through ANOVA when appropriate. Statistically significant was taken when P < 0.05.

## 3. Results

To investigate the regulatory role and potential mechanism of ibrutinib on diabetic I/R injury, we evaluated the effects of ibrutinib on ischemic brain injury, neurological function, inflammatory response, oxidative stress and autophagy in cerebral I/R injury in diabetic mice. In addition, the functional relevance of PI3K/Akt/mTOR signaling pathway in activated autophagy was also investigated.

### 3.1 Ibrutinib exhibits cytoprotective effects against oxidative stress in high glucose-stimulated and H/R injured PC12 cells

**Figure 1.**
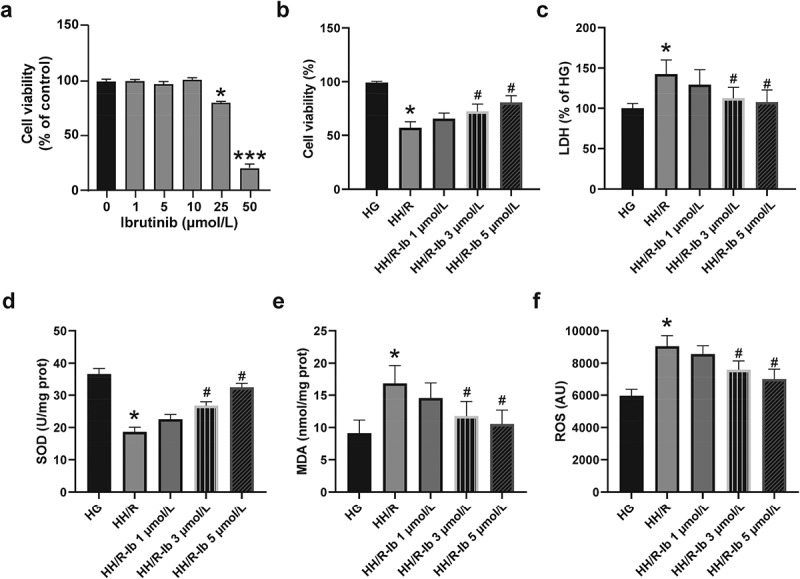
**The cytotoxicity and cytoprotective effects of ibrutinib on PC12 cells without or with high glucose-stimulation and H/R injury**. (a) PC12 cells were incubated with ibrutinib at the final concentrations from 1 μmol/L to 5 μmol/L, and cell viability was measured by MTT assays. (b) The cell viability of the PC12 cells with high glucose and H/R-induced injury. (c) The cytotoxicity was determined by LDH release. Assessment of (d) SOD, (e) MDA, and (f) ROS levels in high-glucose and H/R-induced PC12 cells. Data presented as mean SD, n = 8 replicates/dose. *P < 0.05, ***P < 0.001 *vs*. the HG group or vehicle group, ^#^*P <* 0.05 *vs*. the HH/R group

To explore the treatment effects of ibrutinib on H/R injury in diabetic state, we tested whether ibrutinib has toxicity against PC12 cells. Normal PC12 cells were pretreated with Vehicle (1% DMSO) or ibrutinib at the final concentration of 1, 5, 10, 25, or 50 μmol/L for 24 h. As the cell viability changes are shown in Figure 1a, MTT assays exhibited no significant toxicity against PC12 cells within the final incubation concentration of ibrutinib at 10 μmol (10 μmol Ibrutinib *vs*. Vehicle, *P* > 0.05).

Based on the results above and the published research [23], the non-cytotoxic concentrations (1 μmol/L, 3 μmol/L, and 5 μmol/L) of ibrutinib were selected for evaluating the cytoprotective effects of ibrutinib on high glucose-stimulated and H/R injured PC12 cells. Firstly, the PC12 cells were cultured in the high-glucose medium for 24 h and and treated with hypoxia for 3 h as well as reoxygenation for another 6 h (HH/R). As showed in Figure 1b, the cell viability of PC12 cells was reduced (HH/R *vs*. HG, *P* < 0.05). Higher than 3 μmol/L concentration of ibrutinib improved the viability of HH/R impaired PC12 cells and showed a dose-dependent manner (HH/R-Ib 3 μmol/L, and HH/R-Ib 5 μmol/L *vs*. HH/R, both *P <* 0.05) (Figure 1b). The LDH assay results showed in Figure 1c were correlated with the change of cell viability of PC12 cells. High glucose stimulation and H/R injury significantly increased the LDH extracellular activity in the HH/R group (HH/R *vs*. HG, *P <* 0.05), and when PC12 cells were pretreated with higher than 3 μmol/L ibrutinib, cellular LDH activities were increased dose-dependently (HH/R-Ib 3 μmol/L, and HH/R-Ib 5 μmol/L *vs*. HH/R, both *P <* 0.05) (Figure 1c).

SOD and MDA levels are widely used indicators of oxidative stress in cells, and ROS is the primary cause of cell damage after hypoxia [24]. To further explore the effects of ibrutinib on oxidative stress in PC12 cells, the MDA content, SOD activity as well as ROS content were all measured. After high glucose stimulation and H/R injury, the SOD activity (Figure 1d) was reduced, while the MDA (Figure 1e) and ROS (Figure 1f) levels were relatively increased (HH/R *vs*. HG, all *P <* 0.05). Moreover, the SOD levels (Figure 1c) increased, and the MDA (Figure 1d) and ROS (Figure 1e) levels dose-dependently decreased when the PC12 cells were incubated with 3 μmol/L and 5 μmol/L of ibrutinib compared with the HH/R group (HH/R-Ib 3 μmol/L, and HH/R-Ib 5 μmol/L *vs*. HH/R, both *P <* 0.05).

### 3.2 Effects of ibrutinib treatment on diabetes-related characters in cerebral I/R injured diabetic mice

**Figure 2.**
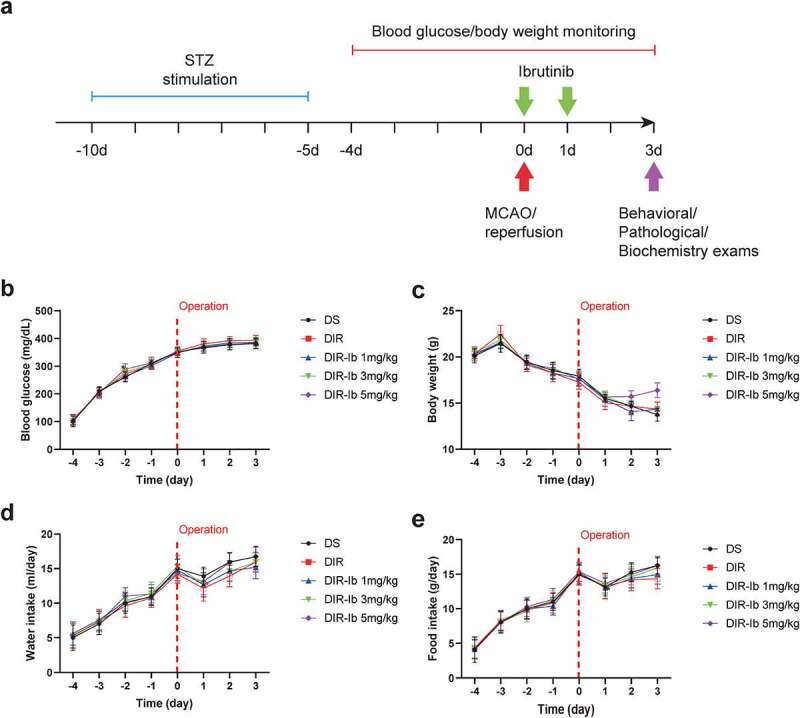
**Blood glucose, body weight, the amount of water/food intake in STZ induced diabetic mice before and after ibrutinib treatment**. Experimental design and timeline (a). Blood glucose level (b) and body weight (c) of STZ induced diabetic mice were monitored five days before and three days after MCAO surgery. Water (d) and food intake (e) of STZ induced diabetic mice were observed five days before and three days after I/R injury. All results presented as mean SD, n = 6/group

As the *in vivo* experimental design showed in Figure 2a, the normal mice were treated intraperitoneally with streptozotocin (STZ) for 5 consecutive days, and the blood glucose level, body weight and the amount of water/food intake were recorded every day until surgery. Then the STZ induced mice were randomly assigned into DS, DIR, three DIR-Ib groups received ibrutinib at the doses of 1 mg/kg, 3 mg/kg and 5 mg/kg. Treatment of Ibrutinib at all three doses were performed twice, once immediately after reperfusion, once 24 h after reperfusion. At the experiment endpoint, mice were sacrificed for pathological characterization and biochemistry exams after evaluating neurological deficits.

The blood glucose levels of each group are shown in Figure 2b. After 3 days of STZ stimulation, blood glucose levels in each group met the criteria of diabetes (≥250 mg/dL) and continued until 3 days after I/R injury which might attribute to both the persistence of STZ and postoperative stress. Ibrutinib treatment exhibited no obvious effects on the developed high blood glucose level after I/R injury (Figure 2b), while the body weight was significantly decreased over time, especially after I/R injury, which is also considered to be the combined effects of STZ and operation. Surprisingly, treatment of ibrutinib at 5 mg/kg slightly inhibited the weight loss at day 3 after I/R injury, but the difference was not significant (Figure 2c). Comparing to the increased water (Figure 2d) and food (Figure 2e) intake of the diabetic model mice which received the STZ stimulation, those of each group were all markedly reduced at day 1 after I/R injury, which might be caused by anesthesia and postoperative pain, and gradually recovered on the following 2 days.

### 3.3 Neuroprotective effects of ibrutinib against cerebral I/R injury in diabetic mice

**Figure 3.**
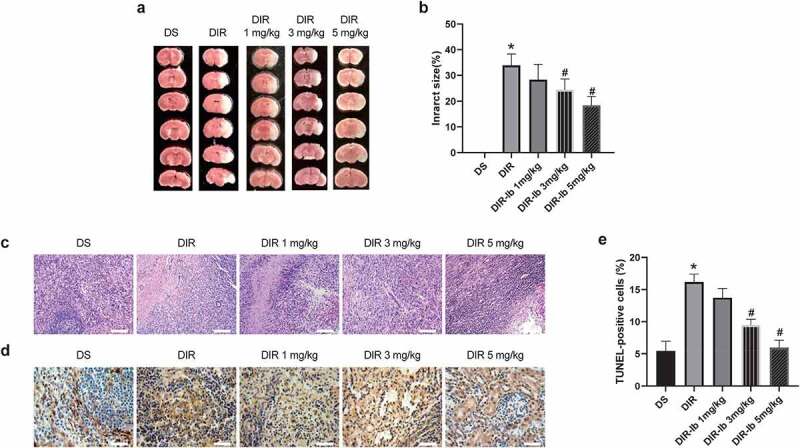
**Ibrutinib protected against cerebral I/R injury in diabetic mice dose-dependently**. (a-b) The TTC staining and infarct size of brain tissue were shown, and (c) The H&E staining was performed for pathological changes (Scale bar: 100 μm). (d-e) Apoptotic cells were accessed by TUNEL assays and brown color indicates apoptotic cells (Scale bar: 50 μm). All results presented as mean SD, n = 6/group. *P < 0.05 *vs*. the DS group, ^#^*P <* 0.05 *vs*. the DIR group

Previous publishment reported that ibrutinib is effective in alleviating ischemic brain injury [16]. To investigate whether ibrutinib exhibits neuroprotective effects on cerebral I/R injured diabetic mice, we further observed the brain tissue injury in each group after ibrutinib treatment. The DIR group’s cerebral infarct volume was notably increased compared with the DS group at day 3 after reperfusion (DIR *vs*. DS, *P <* 0.05) (Figure 3a-b). Administration of ibrutinib distinctly decreased the infarct size dose-dependently compared with that in DIR group, and ibrutinib exerted significantly protective effects at dosages up to 3 mg/kg (DIR-Ib 3 mg/kg, and DIR-Ib 5 mg/kg *vs*. DIR, both *P <* 0.05) (Figure 3a-b). The pathological damage of brain tissue was observed by H&E staining (Figure 3c). The positive cells were intact and abundant, and no infiltration of inflammatory cells in sham group. Nevertheless, we observed severely damaged neuronal structures, unevenly distributed cytoplasm, formed vacuoles, and condensed in the DIR group. In DIR-Ib 3 mg/kg and DIR-Ib 5 mg/kg groups, most parts of the brain structures were restored to normal, the vast majority of neurons appeared to be integrated, and the number of cell nuclei was ameliorated compared with the DIR group. Apoptosis was evaluated using the TUNEL assay in brain tissue. As the results showed in Figure 3d-e, the apoptotic cells significantly increased in I/R injured diabetic mice (DIR *vs*. DS, *P <* 0 05). While treated with ibrutinib at the doses of 3 mg/kg and 5 mg/kg, apoptosis cells were obviously decreased compared with the DIR group (DIR-Ib 3 mg/kg, and DIR-Ib 5 mg/kg *vs*. DIR, both *P <* 0.05).

### 3.4 Ibrutinib dose-dependently improved neurological dysfunction in I/R injured diabetic mice

**Figure 4.**
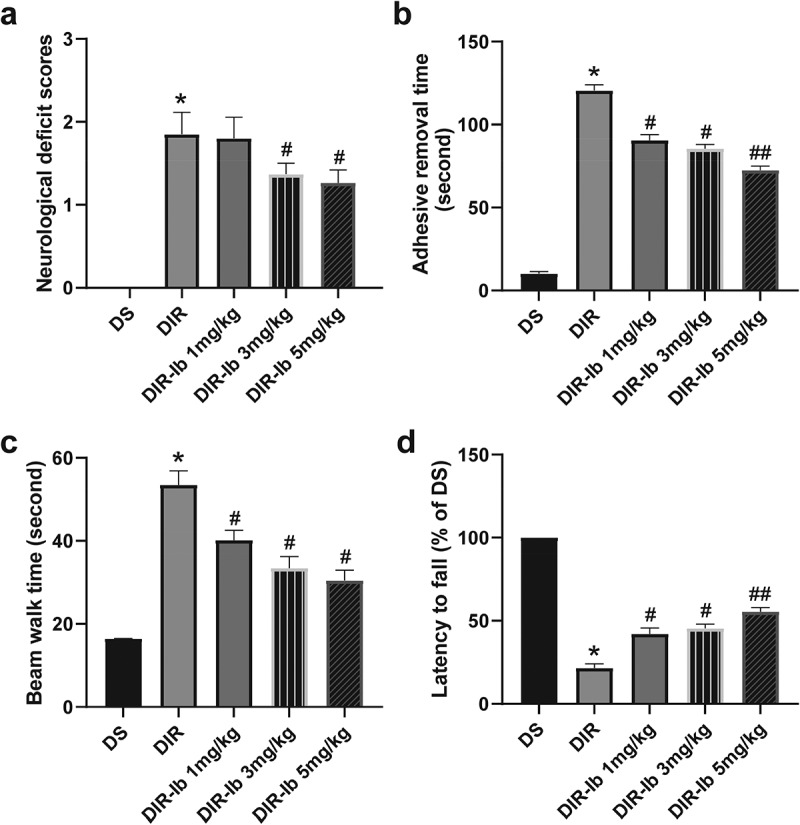
**Behavioral deficits in I/R injured diabetic mice were ameliorated by ibrutinib in a dose-dependent manner**. (a) Neurological deficit assessment, (b) forepaw adhesive removal tests, (c) beam walk tests, and (d) rotarod tests showed improvement with ibrutinib in a slightly dose-dependent manner three days after I/R injury. Data presented as mean SD, n = 6/group. *P < 0.05 *vs*. the DS group, #*P <* 0.05 *vs*. the DIR group

To determine neurological dysfunction in I/R injured diabetic mice, several behavioral tests were chosen to evaluate neurological deficits, sensorimotor, balance, and motor function one day after I/R injury. As results shown in Figure 4a, the neurological deficiency in the DIR group were much more severe in I/R injured diabetic mice (DIR *vs*. DS, *P <* 0.05). In the DIR-Ib 3 mg/kg and DIR-Ib 5 mg/kg groups, the neurological deficit scores were ameliorated compared with the DIR group dose-dependently (DIR-Ib 3 mg/kg, and DIR-Ib 5 mg/kg *vs*. DIR, *P <* 0.05), demonstrating improved neurological function (Figure 4a). The adhesive removal test was performed to evaluate the sensorimotor function. Mice in DIR group showed increased tape removal time than mice in DS group, and the 3 groups treated with ibrutinib showed decreased tape removal time dose-dependently compared with mice in DIR group (DIR-Ib 1 mg/kg, DIR-Ib 3 mg/kg, and DIR-Ib 5 mg/kg *vs*. DIR, *P <* 0.05), demonstrating improved sensorimotor deficits (Figure 4b).

The beam walk test was performed to determine behavioral balance, and the time each mouse took crossing the beam was measured. Mice in DIR group showed increased beam walk time compared with mice in DS group, and the 3 groups treated with ibrutinib walked significantly faster through the beam dose-dependently compared with mice in DIR group (DIR-Ib 1 mg/kg, DIR-Ib 3 mg/kg, and DIR-Ib 5 mg/kg *vs*. DIR, *P <* 0.05), demonstrating improved balance (Figure 4c). We used the rotarod test for motor coordination. Mice in the DIR group demonstrated extreme impairment in the ability to stay on the rod compared with mice without cerebral ischemic, and mice the DIR-Ib 3 mg/kg and DIR-Ib 5 mg/kg groups showed increased latency to fall from the rod, demonstrating improved motor coordination (DIR-Ib 3 mg/kg, and DIR-Ib 5 mg/kg *vs*. DIR, *P <* 0.05) (Figure 4d).

### 3.5 Ibrutinib ameliorated I/R-induced inflammatory response and oxidative stress in I/R injured diabetic mice

**Figure 5.**
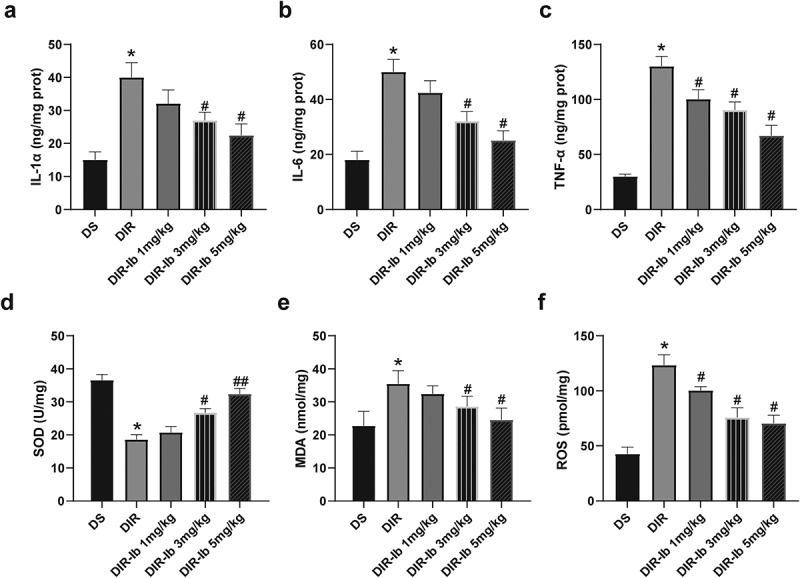
**Ibrutinib decreased oxidative stress and inflammatory response in I/R injured brain tissue of diabetic mice**. The injured lateral hemispheres were processed for detection of (a) IL-1β, (b) IL-6, and (c) TNF-α concentrations. Brain tissues were processed for detection of (d) SOD, (e) MDA, and (f) ROS levels. Data presented as mean SD, n = 8/group. *P < 0.05 *vs*. the DS group, #*P <* 0.05 *vs*. the DIR group

Since it has been reported that ibrutinib associated with significant neuroimmunological changes and oxidative stress, we further measured IL-1β, IL-6, and TNF-α levels of the injured lateral hemisphere in each group. It is observed that IL-1β (Figure 5a), IL-6 (Figure 5b), and TNF-α (Figure 5c) levels were significantly increased in the I/R injured diabetic mice (DIR *vs*. DS, *P <* 0.05). The levels of IL-1β (Figure 5a) and IL-6 (Figure 5b) were markedly attenuated by 3 mg/kg and 5 mg/kg dosage of ibrutinib dose-dependently (DIR-Ib 3 mg/kg, and DIR-Ib 5 mg/kg *vs*. DIR, *P <* 0.05), while the level of TNF-α was attenuated in the DIR-Ib 1 mg/kg, DIR-Ib 3 mg/kg, and DIR-Ib 5 mg/kg group (DIR-Ib 1 mg/kg, DIR-Ib 3 mg/kg, and DIR-Ib 5 mg/kg *vs*. DIR, all *P <* 0.05) (Figure 5c). As for oxidative stress, SOD levels (Figure 5d) were obviously decreased, and the MDA (Figure 5e) and ROS (Figure 5f) levels were significantly increased in the DIR group compared the DS group (DIR *vs*. DS, *P <* 0.05). Treatment of ibrutinib at 3 mg/kg and 5 mg/kg both significantly increased the SOD levels (Figure 5d) and reduced MDA (Figure 5e) and ROS (Figure 5f) content dose-dependently compared to the DIR group (DIR-Ib 3 mg/kg, and DIR-Ib 5 mg/kg *vs*. DIR, both *P <* 0.05). The above results collectively suggested that in I/R injured diabetic mice, ibrutinib could effectively alleviate the inflammatory response and oxidative stress.

### 3.6 Ibrutinib improved autophagy via activating the PI3K/Akt/mTOR pathway in I/R injured diabetic mice

**Figure 6.**
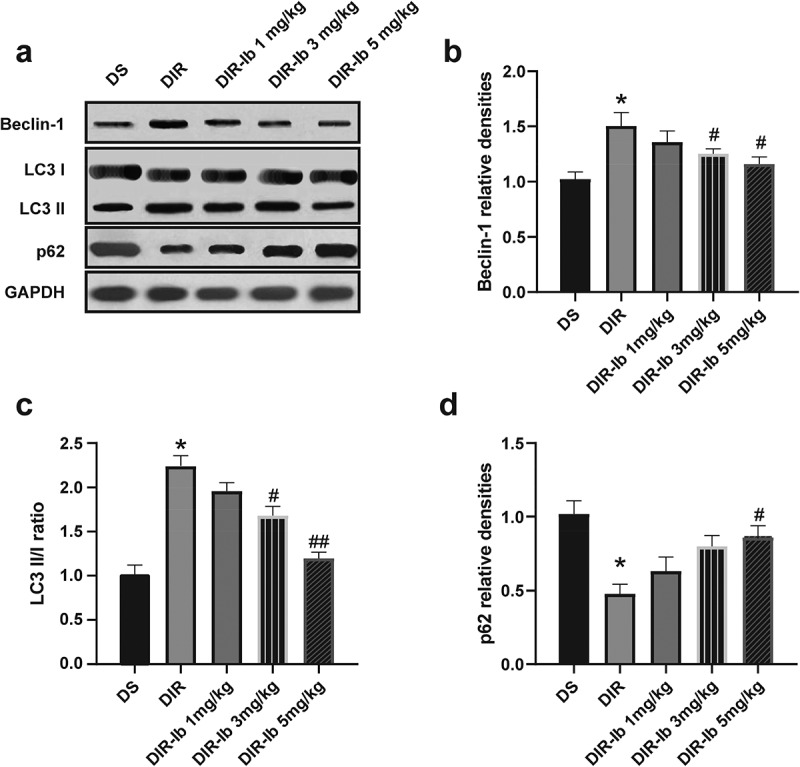
**Effects of Ibrutinib on the autophagy in I/R injured diabetic mice**. (a) Representative western blot image of beclin-1, LC3I, LC3II, and p62. Quantification of (b) becilin-1, (c) LC3II/I, and (d) p62 expression. Data are presented as mean SD, n = 6/group. *P < 0.05 *vs*. the DS group and #*P <* 0.05 *vs*. the DIR group

To confirm alterations in autophagy, the autophagic indicator LC3, beclin-1, and p62 were all measured in each group’s I/R injured lateral hippocampus. LC3 is a generally used marker for autophagosome, and LC3-I/LC3-II conversion represents increased autophagy activation while the degradation of p62 at the late step of the autophagy process serves as a marker for autophagic flux. Beclin-1 is an initiator of autophagy, and p62 is a selective autophagy receptor that reflects the inhibition of autophagy. The expressions of becilin-1 (Figure 6a and 6b), LC3 II and L3 I (Figure 6a and 6c), and p62 (Figure 6a and 6d) were detected by Western blot. I/R increased beclin-1 and LC3 II/I levels, in the meantime, decreased the p62 level in diabetic mice (DIR *vs*. DS, *P <* 0.05). The levels of beclin-1 and LC3 II/I were significantly decreased after administration of 3 mg/kg and 5 mg/kg ibrutinib (DIR-Ib 3 mg/kg, and DIR-Ib 5 mg/kg *vs*. DIR, *P <* 0.05), and p62 was markedly increased after administration of 5 mg/kg ibrutinib compared with the DIR group (DIR-Ib 5 mg/kg *vs*. DIR, *P <* 0.05).

**Figure 7.**
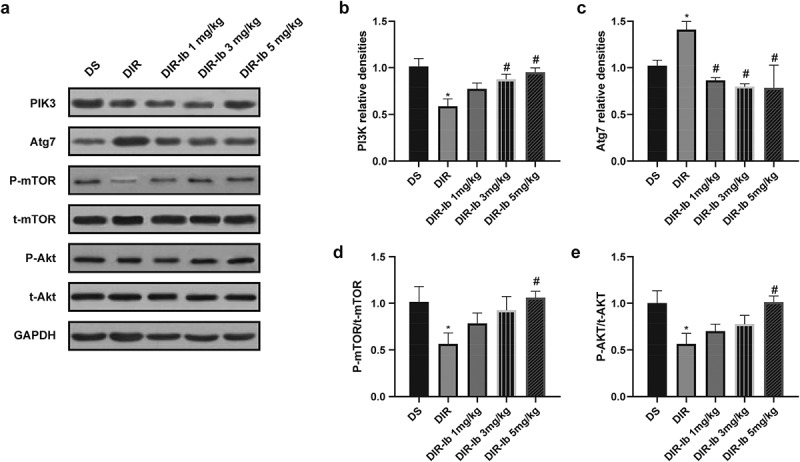
**Ibrutinib modulates the PI3K/Akt pathway in I/R injured diabetic mice**. (a) Representative western blot image of PI3K, Atg7, p-mTOR, t-mTOR, p-Akt, and t-Akt. Quantification of (b) PI3K, (c) Atg7, (d) p-mTOR/t-mTOR, (e) p-Akt/t-Akt levels in each group. Data presented as mean SD, n = 6/group. *P < 0.05 *vs*. the DS group and #*P <* 0.05 *vs*. the DIR group

To address the impact of ibrutinib on the PI3K/Akt/mTOR pathway, the protein expression of PI3K, t-AKT, p-AKT, t-mTOR and p-mTOR were measured in the I/R injured lateral hemispheres. The PI3K level was decreased in the DIR group (DIR *vs*. DS, *P <* 0.05), whereas 3 mg/kg and 5 mg/kg ibrutinib administration reversed the change (DIR-Ib 3 mg/kg, and DIR-Ib 5 mg/kg *vs*. DIR, *P <* 0.05) (Figure 7a-b). The Atg7 expression was increased in the I/R injured diabetic mice (DIR *vs*. DS, *P <* 0.05), while in the ibrutinib treated groups, the expression of Atg7 was reduced (DIR-Ib 1 mg/kg, DIR-Ib 3 mg/kg, and DIR-Ib 5 mg/kg *vs*. DIR, *P <* 0.05) (Figure 7a-bandFigure 7c). The proportion of phosphorylated mTOR (p-mTOR/t-mTOR) in the DIR group were significantly decreased (DIR *vs*. DS, *P <* 0.05), whereas 5 mg/kg ibrutinib administration reversed this change (DIR-Ib 5 mg/kg *vs*. DIR, *P <* 0.05) (Figure 7a-d). The proportion of phosphorylated Akt (p-mAkt/t-mAkt) in the DIR group was significantly decreased (DIR *vs*. DS, *P <* 0.05) and was also reversed in the DIR-Ib 5 mg/kg group (DIR-Ib 5 mg/kg *vs*. DIR, *P <* 0.05) (Figure 7a-e).

## 4. Discussion

In this research, we have certified that BTK inhibition by ibrutinib, an effective inhibitor that could efficiently penetrate the blood-brain barrier, showed neuroprotective effects in cerebral I/R injured diabetic mice. Ibrutinib significantly increased cell viability and repressed oxidative stress response in the high glucose-stimulated PC12 cells subjected to H/R induced injury. In the I/R injured diabetic mice, ibrutinib reduced the infarct size of the brain, improved pathological changes, ameliorated neurological deficit, suppressed oxidative stress and improved autophagy through PI3K/AKT/mTOR pathway when in the dosage of 3 mg/kg and 5 mg/kg. Nevertheless, twice the administration of ibrutinib at the dosages of whether 1 mg/kg, 3 mg/kg, or 5 mg/kg after I/R injury, showed no therapeutic effects on the major characteristics of diabetes. We surmised that these outcomes were related to multiple factors. First, even though the previous study indicated that BTK inhibition could reduce the incidence of type 1 diabetes [17], there is no evidence of ibrutinib being a treatment of developed diabetes. Second, as a long-term inducer of diabetes, STZ induced pathoglycemia could get progressively worse over four weeks after five-day administration [25]. Third, MCAO surgery may cause postoperative stressed hyperglycemia [26]. Also, accompanying postoperative pain and dyskinesia may lead to aberrant water and food intake. This is the first research to investigate the effects of ibrutinib in I/R injured diabetic model, and the underlying mechanisms of ibrutinib in I/R injury complicated diabetes could be potential guidance for further clinical studies.

I/R injury is a major complication occurring with post-stroke treatment, and currently, there is no practical approach in clinical use. Diabetes is a critical risk factor for the occurrence of ischemic stroke. Studies suggested that diabetes could increase the incidence of ischemic stroke by a factor of 1.8 to 6 times, and diabetic patients are exceptionally vulnerable to ischemic stroke. After the restoration of blood flow, ischemic stroke patients with diabetes are more frequently suffering from I/R injury, and it depicts a worse prognosis and higher mortality rate in diabetic stroke patients than ischemic stroke patients without diabetes [27].

Oxidative stress is convincingly a critical pathogenetic factor for diabetic complications. Oxidative damage is a direct consequence of ROS production, and ROS could also inhibit the endothelial nitric oxide synthase, and further increase the production of peroxynitrite anion, which is a strong oxidant that based on the interaction between SOD and nitric oxide, thus exacerbate the injury [28]. It indicated the increased oxidative stress and the following secondary injury are possibly why diabetic patients are more susceptible to cerebral I/R injury. Our results suggested that increased ROS generation and excessive products of lipid peroxidation, along with a lack of antioxidants in the ischemic hemispheres of I/R injured mice, led to severe brain damage.

Diabetes is associated with autophagy dysfunction, which could be one of the potential pathogenetic mechanisms. Autophagy is a highly dynamic process, depending on the complicated turnover and activation of signaling cascades. Autophagy can be further induced by several stimulants, including ROS aggregates, hyperglycemia, amongst others [11]. When the stress stimulus is sustained and chronic, sustained autophagy activation develops into autophagy deficiency, which is implicated in the progression of diabetes [29]. Evidence had displayed that autophagy is impaired in diabetes [30]. Autophagy has been demonstrated to play an essential role in cerebral I/R injury progression [31]. I/R impels rapid ROS production in the normal state, which can directly increase autophagosome formation through oxidative modification and enhance autophagy [32]. The PI3K/Akt/mTOR pathway negatively regulated autophagy [15]. LC3, P62, and Beclin-1 are typical biomarkers for autophagy. Beclin-1 is a core component of the Beclin-1/PI3K-III complex and involved in autophagy activation. LC3 and p62 are essential autophagic proteins. During autophagy, LC3 I is converted into LC3 II, thus, the LC II/ I represents the extent of autophagy [33]. p62 is an autophagy substrate, the clearance of p62 indicates autophagy activation. Studies showed that ibrutinib induced autophagic cell death by suppressing the PI3K/Akt/mTOR signaling pathway [34]. Our study found autophagy was normally activated after I/R injury sufficient in diabetes state, and ibrutinib regulated autophagy, which might be one of the mechanisms that ibrutinib attenuate cerebral I/R injury. We need to further explore whether there is a robust relationship between this phenomenon and I/R injury repair in subsequent studies.

## 5. Conclusion

Our results collectively revealed that ibrutinib could effectively ameliorate cerebral ischemia/reperfusion injury via ameliorating inflammatory response, oxidative stress, and improving autophagy through PI3K/Akt/mTOR signaling pathway in diabetic mice.

## Data Availability

All data generated or analyzed during this study are included in this article.

## Highlights

- Ibrutinib improved the PC12 cell viability with H/R injury in the high glucose
- Ibrutinib exerted neuroprotective effects against diabetic cerebral I/R injury
- Ibrutinib treatment improved autophagy through the PI3K/Akt/mTOR pathway
- Ibrutinib ameliorated cerebral ischemia/reperfusion injury in diabetic mice
